# Biological properties and therapeutic effects of plant-derived nanovesicles

**DOI:** 10.1515/med-2020-0160

**Published:** 2020-11-09

**Authors:** Sante Di Gioia, Md Niamat Hossain, Massimo Conese

**Affiliations:** Department of Medical and Surgical Sciences, University of Foggia, 71122 Foggia, Italy; Laboratory of Experimental and Regenerative Medicine, Department of Medical and Surgical Sciences, University of Foggia, Foggia, Italy

**Keywords:** exosome-like nanoparticles, antitumoral, miRNAs, drug delivery, inflammatory bowel disease

## Abstract

Exosomes-like nanoparticles can be released by a variety of plants and vegetables. The relevance of plant-derived nanovesicles (PDNVs) in interspecies communication is derived from their content in biomolecules (lipids, proteins, and miRNAs), absence of toxicity, easy internalization by mammalian cells, as well as for their anti-inflammatory, immunomodulatory, and regenerative properties. Due to these interesting features, we review here their potential application in the treatment of inflammatory bowel disease (IBD), liver diseases, and cancer as well as their potentiality as drug carriers. Current evidence indicate that PDNVs can improve the disease state at the level of intestine in IBD mouse models by affecting inflammation and promoting prohealing effects. While few reports suggest that anticancer effects can be derived from antiproliferative and immunomodulatory properties of PDNVs, other studies have shown that PDNVs can be used as effective delivery systems for small molecule agents and nucleic acids with therapeutic effects (siRNAs, miRNAs, and DNAs). Finally, since PDNVs are characterized by a proven stability in the gastrointestinal tract, they have been considered as promising delivery systems for natural products contained therein and drugs (including nucleic acids) via the oral route.

## 1 Introduction

Natural products and their derivatives have been widely used throughout the human history, and today, they constitutes a large part of the pharmaceutical market [1,2]. For years, pharmaceuticals companies have not paid due attention to these classes of compounds for many reasons, such as the wrong idea that natural products are only useful as a restricted class of drugs, e.g., antibiotics: natural products had huge success in the post-World War II era as antibiotics, and the two terms have become synonymous [1]. Generally, large pharmaceutical companies have focused their attention on screening synthetic compound libraries for drug discovery, whereas small companies have started to explore the role of natural compounds against diseases such as cancer, microbial infection, and inflammatory processes [3,4]. When a potential therapeutic application is considered, one of the biggest issues that make the use of natural compounds quite challenging is their low bioavailability [4]. For instance, it has been calculated that the administration of curcumin orally requires doses of 3.6 g/day to reach serum levels of 11.1 nmol/L, and subjects who take lower doses of curcumin did not have detectable plasma levels [5,6]. Similar results were observed with other natural compounds such as polyphenols and flavonoids [7,8]. In this context, the use of nanoparticles can enhance the efficacy of natural products in disease treatment by increasing their bioavailability. The benefits of nanoparticles are not just a “size-matter” but something more complicated. Indeed, it is well recognized that at a nanoscale level, particles can acquire unique properties. Basically, nanodelivery systems might be useful to overcome the limitations of the traditional natural compounds administration because of the following reasons:

1. Nanoparticles improve the solubility of natural compounds [9].
2. Nanoparticles could target the natural products to specific organ, which improves the selectivity, drug delivery, efficacy, and safety and thereby reduces dose and increases the patient compliance.
3. They appear to be able to deliver high concentrations of drugs to disease sites because of their unique size and high loading capacities.
4. Delivering the drug in small particle size enhances the entire surface area of the drugs, therefore allocating quicker dissolution in the blood.
5. Nanoparticles show enhanced permeation and retention (EPR) effect, i.e., enhanced permeation through the barriers because of the small size and retention due to poor lymphatic drainage such as in tumor [10].
6. Nanoparticles exhibit passive targeting to the disease site of action without the addition of any particular ligand moiety.
7. Due to the previous properties, nanoparticle application decreases the side effects.

There are different types of synthetic nanoparticles used for drug delivery, such as polymer nanoparticles, solid lipid nanoparticles (SLNs), crystal nanoparticles, liposomes, micelles, and dendrimers. Each of these nanoparticles has its own advantages and disadvantages as a drug delivery vehicle. For example, polymer nanoparticles between 10 and 1,000 nm in diameter can have the characteristics desired for an efficient delivery of molecules [4,11]. Unfortunately, these systems are “artificial” as they are obtained by chemical synthesis, and this poses a strong limitation for their application *in vivo* because synthetic nanoparticles have two major limitations: (1) each of their constituents must be evaluated for potential *in vivo* toxicity before clinical application and (2) the production scale is limited.

To overcome these limitations, many research groups focused their attention in developing green, sustainable and biocompatible materials for the delivery of bioactive compounds within pharmaceutical and medical industries. Basically, plant-derived nanovesicles (PDNVs) would be excellent candidates for the delivery of therapeutic agents (e.g., anticancerous drugs, small interfering RNAs (siRNAs), microRNAs (miRNAs)), or poorly soluble natural compounds (e.g., curcumin) [12]. Among the various PDNVs, exosomes have gained attention as a potential nanodelivery system as they are characterized by various desirable properties such as small size, biocompatibility, and high stability. According to the International Society for Extracellular Vesicles (ISEV; www.isev.org), there are three main extracellular vesicles (EVs): exosomes, ranging from 30 to 120 nm and that are produced through the endocytic pathway; microvesicles (MVs), 100–1,000 nm vesicles, that are released from the plasma membrane through outward protrusion or budding; and apoptotic bodies, 1–6 µm in diameter, derived from cells undergoing apoptosis (see Vesiclepedia, www.microvesicles.org and ref. [13]). Recently, ISEV has reviewed many features of EVs, for examples, subtypes should be defined by physical and biochemical characteristics and/or conditions/sources rather than on conventional nomenclature [14]. However, since these recommendations are still to be accepted universally, we refer to terms referred earlier.

**Figure 1:**
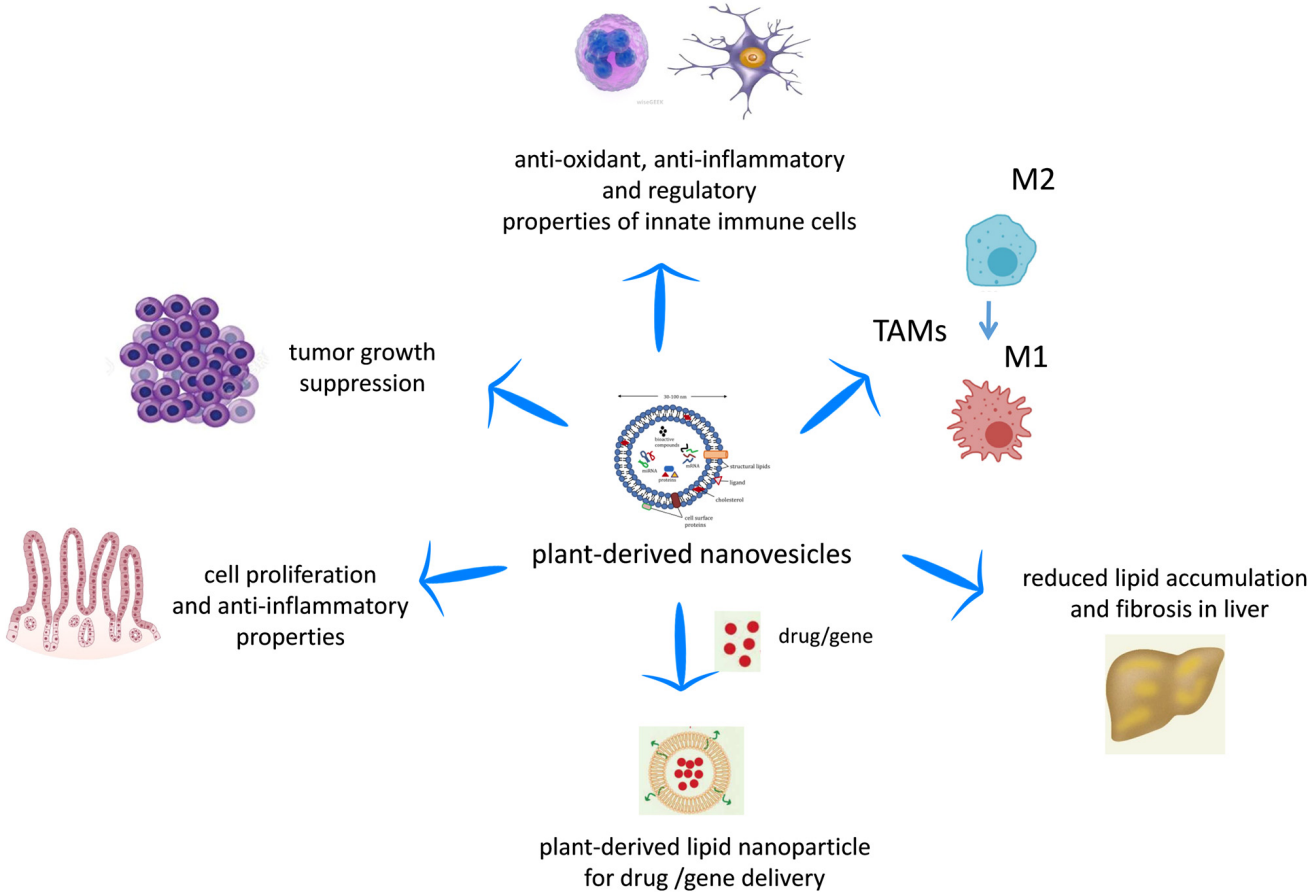
Biological properties of PDNVs. PDNVs can regulate *in vitro* and *in vivo* the function of macrophages and dendritic cells by inducing anti-inflammatory and regulatory functions, as well as shifting tumor-associated macrophages (TAMs) from a M2 to a M1 phenotype. PDNVs have been demonstrated to participate in intestinal tissue homeostasis in *in vivo* animal models and have validated functions against inflammation-related diseases and cancers. Finally, the efficacy of PDNVs for gene or drug delivery has been shown.

For many years, researchers had been guided by the outdated idea that exosomes are waste products obtained from the shedding of plasma membranes, whereas exosome vesicles cargo is composed of proteins, lipids, and nucleic acids. All of these “cargo” biomolecules accumulate inside exosomes and are wrapped by the phospholipid bilayer. This structure allows them to take part in processes such as intercellular communication, exchange of materials with other cells, elimination of unwanted products from cells, and immune surveillance [15,16]. Growing evidence show EVs (MVs along with exosomes) participate in various cell signaling process and are likely involved in pathophysiological processes such as cardioprotection [17] and cancer [18]. Currently, there is evidence that PDNVs may be involved not only in plant–cell communication but also in interspecies communication between plants and animals [19]. For example, a plant-derived miRNA such as miR-168 has been reported to enter the circulation of rice-fed mice enclosed in vesicles and to modulate the expression of target genes [20]. Besides, exosomes found in cell culture medium and biological fluids such as urine, saliva, breast milk, plasma, and cerebrospinal fluid [21], as well as matrix-bound nanovesicles (MBVs), embedded within biological scaffold composed of extracellular matrix (ECM), have been identified [22,23]. Since they are localized to collagen fibrils, likely anchoring via adhesion molecules, MBVs have been isolated by enzymatic digestion of ECM bioscaffolds obtained from urinary bladder matrix, small intestinal submucosa, and dermis [22,23]. Although MBVs share features in common with exosomes and microvesicles, such as their size (10–1,000 nm) and the presence of miRNA cargoes, the lack of identifiable surface markers (such as CD63, CD81, CD9, and Hsp70) and unique nucleic acid and protein cargoes suggest that they represent a different population of signaling vesicles [22,23,24]. MBVs and their miRNA cargoes can recapitulate many of their parent ECM’s effects, including the promotion of a regulatory macrophage phenotype, thus facilitating tissue repair [22]. In addition, MBVs have been shown to positively regulate primary neuron survival and growth [25] as well as to reduce proinflammatory, neurotoxic glial signaling enhancing healing responses in the retina and the optic nerve [26]. Although PDNVs were initially conceived as more similar in structure and function to mammalian exosomes than MBVs [27], they are quite heterogeneous in size (see below) and may have distinct features from mammalian exosomes and MBVs. However, as outlined briefly earlier, the field of EVs is continuously evolving and we may have to deal with new concepts about them in the close future [14]. According to the recent literature, PDNVs, the focus of this review, display many biological properties, which are illustrated in the following sections (Figure 1).

PDNVs display many properties that make them suitable for clinical applications, including a relatively high internalization rate, low immunogenicity, stability in the gastrointestinal (GI) tract, and the ability to overcome the blood–brain barrier but not the placental barrier [28]. Since the GI tract is the first tissue receiving EVs from edible fruits and plants, many researchers focused their attention onto EVs biological functions on the intestinal barrier. These studies have shown that these exosome-like plant-derived EVs can be used for improving inflammatory bowel disease prevention and treatment by blocking damaging factors and promoting healing factors [29,30]. Moreover, the positive action of exosome-like EVs was demonstrated in the liver when injured by alcohol [31]. Also, PDNVs are not cytotoxic and can be considered as novel drug carrier systems used in combination with nucleic acids (siRNAs and miRNAs) as well as with anticancer therapeutics [28,32].

Altogether, today, PDNVs may be considered a real alternative to synthetic nanoparticles for their complex biological properties, therapeutic applications, and drug delivery systems. The aim of this review is to provide up-to-date consideration to PDNVs, highlighting their physicochemical properties, their biological effects, as well as their anti-inflammatory, anti-oxidant, and proregenerative properties. Finally, we will give an insight in the treatment of gastrointestinal/liver diseases and cancer through PDNV-mediated drug delivery.

## 2 Isolation and purification of PDNVs

The isolation of PDNVs is mainly based on differential centrifugation plus density gradient centrifugation. Plants are ground to juice in a mixer, and low-speed centrifugations are used to remove large plant debris and aggregates, whereas ultracentrifugation is used to pellet PDNVs. A “standard protocol” to isolate PDNVs utilizes multiple centrifugation steps (low, medium, and high speeds). Generally, the amount of a raw material (plant or fruit) used to isolate PDNVs is variable, ranging from 2 to 10 g [33,34] to 250 g [31]. Initially, intact cells are removed by low-speed centrifugation (e.g., 1,000 × *g*). The supernatant is then subjected to centrifugal forces in the range of 10,000–20,000 × *g* to remove large debris and intact organelles. This supernatant is then subjected to centrifugation at high speed (100,000–150,000 × *g*). While the stated methodology is relatively straightforward, the type, quantity, and quality of the PDNVs isolated by ultracentrifugation is highly sensitive to multiple parameters, including the g force, the rotor type (fixed angle or swinging bucket), the angle of rotor sedimentation, radius of the centrifugal force, pelleting efficiency (rotor and tube *k*-factors), and solution viscosity [35,36,37]. Moreover, the highest speed used (100,000 × *g* or greater) also sediments other vesicles, proteins, and/or protein/RNA aggregates. Thus, a subsequent sucrose density gradient step is used to separate the PDNVs from contaminants of different densities. Gradient ultracentrifugation requires an extended centrifugation time (1–5 h), but provides a more purified edible plant nanoparticle isolate than ultracentrifugation alone [38]. Other methods such as ultrafiltration and immunoisolation have been implemented for animal-derived exosomes to obtain purer preparations [38,39]; however, they have some drawbacks, including higher costs and have not been extensively used for PDNVs purification.

The yield is variable depending on the plant source and the method used for quantification. Zhang et al. [40] reported a high yield of exosome-like vesicles of 48.5 ± 4.8 mg per 1 kg of ginger. In another study, the production yields of PDNVs are very similar across the fruits and root-derived edible plants, with 100 g of edible plant material producing about 350–450 mg of nanoparticles [41]. In alternative to weighing, the yield of PDNVs can be measured by a zeta-sizer. For instance, among the mushrooms tested, oyster mushroom-derived exosome-like nanoparticles had the lowest yield of 2.3 ± 1.5 × 10^11^/g, whereas white button mushroom-derived PDNVs had the highest yield of 8.1 ± 1.6 × 10^11^/g [42].

Scalability in the production of nanoscale materials is an inherent problem for nanomedicine, for instance, in the case of liposomes that are the more approximate drug vehicle to exosomes and PDNVs. The high costs strongly limit the large-scale production of artificial nanovesicles and mammalian cell-secreted exosomes to be employed in humans as commercial products [28,43]. Although PDNVs can be produced economically [44] and have a significant potential for large-scale production [19,45], due to the high yield as outlined earlier, the field lacks scalable methods to efficiently isolate and assemble PDNVs of uniform size [28].

## 3 Physicochemical and biological properties of PDNVs

PDNVs from edible plants and fruit have been characterized for their physical parameters (size and surface charge), biomolecules content (lipids, protein, and miRNAs), and biological properties. Here, we provide an overview of PDNV characteristics (Table 1).

**Table 1:** Physicochemical and biological properties of plant-derived nanovesicles

Source of plant-derived nanoparticles (PDNVs)	Chemical properties			Physical properties		Biological properties				Ref.
	Lipids	Proteins	RNAs	Size and surface charge (zeta potential)	Structure	Toxicity	Cell uptake	Natural targeting properties	Stability	
Grape exosome-like nanoparticles (GELNs)	98% phospholipids (50% phosphatidic acids), 2% galactolipids	28 proteins Proteins regulating the carbohydrate/lipid metabolism	96 miRNAs	Size	Referred as exosome like		Uptaken by mouse intestinal epithelial cell line CT26			[49]
				380.5 ± 37.47 nm Charge −26.3 ± 8.14 mV						
			
	
Grapefruit-derived nanovesicles (GFDNs)	29% phosphatidylcholine, 46% phosphatidylethanolamine	137 proteins Proteins regulating the carbohydrate/lipid metabolism		Size 210.8 ± 48.62 nm Charge −49.2 to −1.52 mV		Nontoxic to mouse macrophage cell line	Uptaken by mouse intestinal macrophages	Intestinal macrophages of mouse model	Very stable at physiologic temperature (37 °C)	[53]
	Naringin and naringenin									
			
			
Grapefruit-derived nanovesicles (GFDNs)	24% phosphatidylethanolamine, 23% phosphatidylcholine, 13% phosphatidylinositol, 10% diacylglycerol			Size	Multilayer flower-like structures	Nontoxic to A549 and CT26 cells (nonhematopoietic cells)	Uptaken by GL26, A549, SW620, CT26, and 4T1 cells (tumor cell lines)	Splenic and liver cells of mouse model	Very stable at 4 °C for more than one month and a loaded cargo (curcumin)	[32]
				180 to 200 nm						
Ginger-derived nanovesicles (GDNs)	37.03 and 40.41% phosphatidic acid for band 1 and band 2 respectively, 39.93 and 32.88% digalactosyldiacylglycerol for band 1 and band 2 respectively, 16.92 and 19.65% monogalactosyldiacylglycerol for band 1 and band 2, respectively			Size			Uptaken by primary hepatocytes	Hepatocytes are the primary targeted cells	Very stable in stomach-like (pH 2.0) and small intestine-like solutions (pH 6.5)	[31]
				∼386.6 nm for band 1, ∼294.1 nm from band 2						
				Charge						
				−24.6 mV to −29.7 mV						
Ginger-derived nanovesicles (GDNs)	∼25–40% phosphatidic acid, ∼25–40% digalactosyldiacylglycerol, ∼20–30% monogalactosyldiacyglycerol	actin and proteolysis enzymes, membrane channel/transporters	125 different miRNAs (15–27 nt)	Size		Nontoxic to Colon-26 epithelial-like cell lines, RAW 264.7 macrophage-like cell lines		Colon of mouse model	Extremely stable at room temperature over 7 days (band 1 and 2), tolerate freeze/thaw cycles	[44]
				∼292.5 nm form band 1, 232 nm from band 2, 220 nm from band 3.						
				Charge						
				−12 mV at pH 6 for band 1 and 2, −2.1 mV for band 3						
Ginger-derived nanovesicles (GDNs)				Size	Exosome-like	Low cytotoxicity on somatic cell (HEK293), cancer cell (KB)				[68]
				Cushion method: 123.5 nm						
				Pellet method: 124.5 nm						
Ginger-derived nanovesicles (GDNs)				Size			Uptaken by BMDM (bone marrow-derived macrophages)			[33]
				DLS: ∼130 nm						
				SEM: 120–150 nm						
*Citrus limon* L. derived nanovesicles (CDNs)		580 proteins		Size ranged between 50 and 70 nm	Exosome-like		Uptaken by A549 (human lung carcinoma cell line), LAMA84 (chronic myeloid leukemia cell line)	Liver, spleen, and partially by kidneys of mouse model		[55]
*Citrus limon* L. derived nanovesicles (CDNs)			23 nucleotide small RNAs	Size	Round or cup-shaped objects	Nontoxic to MSCs (mesenchymal stromal cells)	Uptaken by MSCs			[66]
				30–100 nm						
*Citrus clementina* fruit juice-derived nanovesicles (CFDNs)		1,018 proteins, 162 proteins associated with transport		Size 75–345 nm						[56]
		Gene								
		Ontology (GO): 71 transmembrane transporters, 53 vesicle-mediated transporters and 50 intracellular transporters								
Edible plant-derived exosome-like nanoparticles (11 fruits and vegetables)	Lipid profile in grape- and grapefruit-derived NVs different from that in ginger and carrot		418 conserved miRNAs, Ginger (*n* = 32), Soybean (*n* = 127)	Size	Round or oval					[61]
				100–1,000 nm						
Edible plant-derived exosome-like nanoparticles (grape, grapefruit, ginger, carrot)			Total RNAs extracted from grape and grapefruit much less abundant than in ginger and carrot.					Intestinal macrophages and stem cells	The size of NVs was altered in stomach-like and intestinal-like conditions in a pH-dependent manner.	[41]
			Grape NVs contain miRNAs that are enriched for miR169 family.							
Exosome-like nanoparticles from coconut water			36 miRNAs	Size						[34]
				coconut water 59.72 nm, milk sample 100.40 nm						
Broccoli-derived nanoparticles (BDNs)	Lipids (sulforaphane)			Size						[64]
				∼18 and 118 nm						
				Charge						
				−39 to −2.6 mV						
Broccoli phytochemicals–coated gold nanoparticles (B-AuNPs)				Size			Uptaken by breast (triple negative) cancer cell lines MDA-MB-231 and prostate cancer cell lines PC-3		Excellent stability in biological fluids (0.5% cysteine, 0.2 M histidine, 0.5% human serum albumin (HSA), 0.5% bovine serum albumin (BSA), and 1% NaCl solutions) at physiological pH (pH 7 and 9)	[65]
				hydrodynamic size as 90 ± 5 nm						
				Charge						
				−29.0 mV						
Wheat-derived nanovesicles (WDNPs)				Size	Exosome-like	Nontoxic to HDF (primary human dermal fibroblast cell line), HUVEC (human umbilical vein endothelial cells), and HaCaT cells (human keratinocyte cell line)				[69]
				Between 40 and 100 nm						
Ginseng-derived nanovesicles (GSNVs)	59.4% digalactosyl monoacylglycerol, 16.8% phosphatidyl ethanolamine, 13.8% ceramide	3,129 proteins		Size	Similar to mammalian-derived extracellular vesicles	Nontoxic to BMDMs (bone marrow-derived macrophages)	Uptaken by BMDMs (bone marrow-derived mouse macrophages)	Liver and spleen of mouse model		[54]
				∼344.8 nm for band 3		B16F10 (mouse melanoma cell line)				
				Charge		4T1 (mouse mammary carcinoma line), HEK293T (human embtyonic kidney cell line)				
				−25.4 mV						
Apple-derived nanoparticles (APNPs)			RNAs ranging in size from 20 to 30 nt and from 50 to 70 nt	Size			Uptaken by Caco.2 cells (intestinal epithelium)		Disappear when boiled or sonicated	[67]
				100–400 nm by nanosizer						
				100–200 nm by eectron microscopy						

### 3.1 Chemical properties

Lipids are key components of the lipid bilayer structures of PDNVs, which have distinct composition from mammalian cell-derived exosomes and artificially synthesized liposomes [46,47,48]. Lipid profiling has ascertained that two major classes of lipids in PDNVs are phospholipids and glycerol lipids, but they lack cholesterol [28]. Ju et al. [49] identified exosome-like nanoparticles from grapes (GELNs) using electron microscopy examination and found they consist of 98% phospholipids, among which approximately 50% has been identified as phosphatidic acids (PA). Differential centrifugation and sucrose gradient ultracentrifugation were used to isolate GELNs and a triple quadrupole mass spectrometer was used to determine their lipid composition. The presence of an extremely small amount (∼2%) of usual plant lipids (e.g., galactolipids, such as digalactosyldiacylglycerol (DGDG) and monogalactosyldiacylglycerol (MGDG)) have also been reported [49]. PA is a cell-signaling lipid with many biological activities, including the activation of the mammalian target of rapamycin (mTOR) and mitogen-activated protein kinase (MAPK) pathways which could explain PDNV action on cell growth and proliferation [50,51]. PA is also involved in vesicular trafficking, secretion, and endocytosis, likely by affecting the cytoskeletal organization [50,51], suggesting its role in the uptake of PDNVs by mammalian cells. More recently, it has been shown that PDNVs (obtained from ginger) are preferentially taken up by *Lactobacillus rhamnosus*, a process mediated by PA, and this alters the composition of gut microbiota. Mass spectrometry (MS) analysis was done to assess the comparative lipid profiles, which showed that PDNVs from ginger are enriched with PA (35.2%) [52].

Another study reported that grapefruit-derived nanovesicles (GFDNs) encompassed higher levels of phosphatidylcholine (PC, 29%) and phosphatidylethanolamine (PE, 46%) [53]. A triple quadrupole tandem mass spectrometer was used to analyze the lipid composition and the data were represented as percentage of total signal of the molecular species determined after normalization of the signals to internal standards of the same lipid class [53]. Similar study with GFDNs revealed the composition as 24% PE, 23% PC, 13% phosphatidylinositol (PI), and only 10% diacylglycerol (DG) [32]. Like the other scientific groups, tandem mass spectrometer similar to the previous study was used to determine the lipid composition. On the other hand, the scenario is different in case of the study of ginger-derived nanoparticles (GDNs) which reported that the lipid composition analyzed by triple quadrupole mass spectrometer encompassed 42% PA, 27% DGDG, and 19% MGDG. In this case the data represented as mol% of the total lipid analyzed [44]. Study with ginseng-derived nanoparticles (GSDNs) by MS revealed that they are comprised of DGMG (59.4%), PE (16.8%) and ceramide (13.8%). Among them DGMG and ceramide are not familiar lipids with other plant derived nanoparticles [54]. As we shall see in the Section 3.4 (“PDNVs as drug carrier”), lipids from PDNVs can be applied as suitable agents for transporting different therapeutic agents.

According to Yang et al. [28], there are only few reports available considering the proteins of PDNVs and also the results are not consistent enough. It is reported that PDNVs have a relatively low protein content in comparison to mammalian cell-derived exosomes and that the protein compositions were different from those of mammalian exosomes [44]. As mentioned by Ju et al. [49], GELNs represented 28 detected proteins by analysis using MS. Conversely, around 137 proteins have been isolated from GFDNs through the MS analysis [53]. Zhang et al. [44] reported that GDNs mostly consist of cytosolic proteins (mainly actins and proteolytic enzymes). A few low-abundance membrane proteins such as membrane channels/transporters (e.g., aquaporin and chloride channels) were also identified and quantified by ultra-performance liquid chromatography tandem mass-spectrometry (UPLC-MS/MS) [44]. Raimondo et al. [55] identified 580 proteins from *Citrus limon* L. juice-derived nanovesicles (CDNs) which were characterized by gel-based approach (GeLC-MS/MS) and liquid chromatography (LC)-MS/MS system. Approximately 57% of these proteins overlapped with those found in mammalian cell-derived exosomes, irrespective of the cell origin. Another similar study identified 1,018 proteins from *Citrus clementina* juice-derived nanovesicles (CFDNs) using MS-based organelle proteomics [56]. In a study on GSDNs, Cao et al. [54] identified 3,129 proteins by analyzing through MS, which were classified using the gene ontology (GO) analysis into three categories: biological process, cellular compartment, and molecular function.

The PDNV content of mRNAs, miRNAs, and noncoding RNAs is similar with that of mammalian cell-derived exosomes [44,57]. miRNAs are a group of molecules that are single-stranded RNAs and small in size containing only 18–24 nucleotides [58]. They play important roles in posttranscriptional regulation in animals and plants [59,60]. PDNVs carried a significant number of miRNAs, as in the case of 125 for GDNs as estimated by deep sequencing [44]. Among them, 124 miRNAs could potentially target and regulate the expression of human genes by binding to their 3′-untranslated regions (3′-UTRs). Ninety-six miRNAs have been reported from GELNs as obtained by the MS analysis [49]. Recently, 418 conserved miRNAs were identified from 11 edible fruit and vegetables (from 32 for ginger EVs to 127 for soybean EVs), which were sequenced by Illumina HiSeq 2,500 platform and identified in miRBase21.0 against known plant mature miRNAs [61]. Bioinformatics analyses were performed to predict functional relationships between plant-derived miRNAs and their potential target genes in the mammalian genome. Interestingly, it was found that these mammalian genes were involved in immune response and cancer-related pathways, the two main functions associated with plant-derived exosomes.

The comparison among different methods of detection of lipids and proteins may explain the differences in detected proteins and lipid composition and also biological properties shown by the various PDNVs.

### 3.2 Physical properties

**Figure 2:**
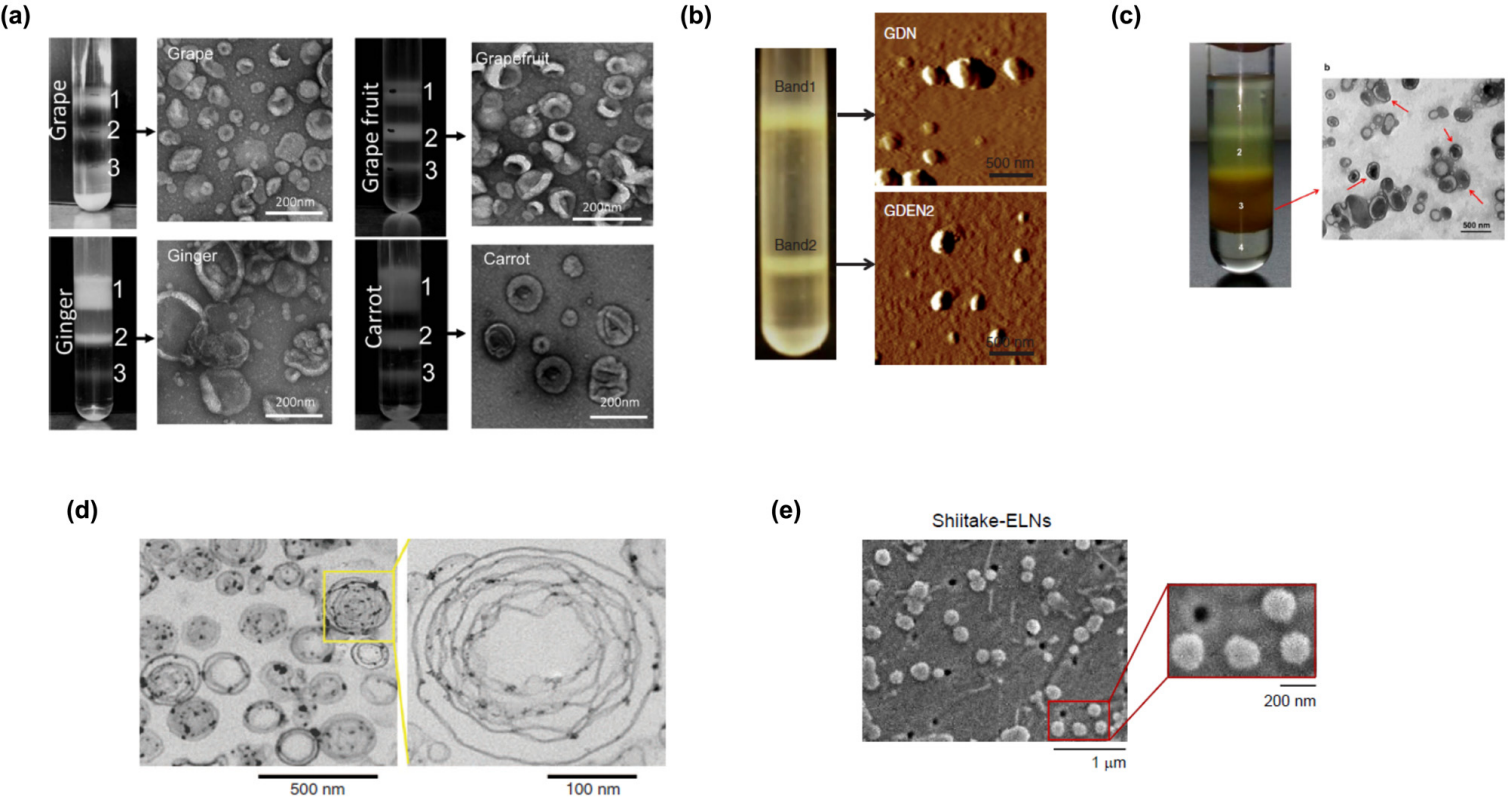
Physical characteristics of PDNVs obtained from different edible plants. (a) Three bands were formed after sucrose gradient ultracentrifugation. NVs from grape, grapefruit, ginger, and carrot from the 30%/45% interface were visualized by the electron microscopy. Reprinted from ref. [41] with permission from John Wiley and Sons. (b) Two bands from sucrose-banded ginger rhizome root-derived samples were formed after gradient ultracentrifugation (left). GDN and GDN2 particles were visualized by AFM (right). From ref. [31]. (c) Ginseng root juice was purified by sucrose gradient ultracentrifugation, and the band from the interface of 45% (band 3) was harvested (left panel) and characterized by TEM (right panel). Adapted from ref. [54]. (d) Grapefruit-derived lipids were analyzed by electron microscopy. Original magnification was 50,000×. A multilayer flower-like structure is observable. Reprinted from ref. [32] with permission from Springer Nature. (e) *Shiitake mushroom*–derived exosome-like NVs under SEM. Main figure: magnification 20,000×, inset: magnification 50,000× (from ref. [42]).

The normal size distribution of PDNVs ranges from 30 to 1,000 nm. Structures smaller than 30 nm are excluded from consideration because of the difficulty of packing lipids inside a strongly curved geometry. Their drug-loading capacity is also correspondingly low [62]. Generally, PDNVs display negative zeta potential value ranging from −100 to around 0 mV, illustrating their mutual repulsion and lacking aggregation tendency [41]. In one of the first reports, PDNVs from grape, grapefruit, ginger, and carrot were characterized as exosomes based on the electron microscopic estimation of a sucrose gradient purified band 2 (Figure 2a), and other determinations [41]. GDNs were identified as band 1 and band 2 by sucrose gradient ultracentrifugation. The average size distribution was ∼292.5 nm for band 1 and 232 nm for band 2. The zeta potential value detected at pH 6 (the pH of the duodenum–jejunum) was −12 mV for both band 1 and band 2 [44]. Atomic force microscopy (AFM) revealed that GDNs are spherical nanoparticles (Figure 2b). Chen et al. [33] found that GDNs were approximately 130 nm in diameter by dynamic light scattering (DLS) and precisely 120–150 by scanning electron microscopy (SEM). Conversely, GDNs had a size distribution between ∼294 and 386 nm by the sucrose density gradient system and a zeta potential value of −25 mV [63]. The size distribution of exosome-like nanoparticles from coconut water (CCDNs) was evaluated by DLS and SEM. These studies revealed that the particles have mean diameters of 60 and 13 nm, respectively [34]. Interestingly, those derived from coconut milk were greater, i.e., around 100 nm. The size distribution of broccoli-derived nanovesicles (BDNs) was evaluated by a nanosizer and affirmed by electron microscopy and ranged from ∼18 to 118 nm. The zeta potential value was also measured, which showed a negative zeta potential value approximately from −39 to −2.6 mV [64]. Another study with broccoli phytochemical–coated gold nanoparticles (B-AuNPs) reported the hydrodynamic size as 90 ± 5 nm by DLS. The zeta potential value was −29.0 mV, which provided the necessary repulsive forces for the particles to remain stable in solution [65]. GFDN-derived lipids resembled multilayer flower-like structures by electron microscopy, with a size distribution by the DLS analysis ranging from 180 to 200 nm in diameter [32] (Figure 2d). In the previous study, Cao et al. [54] obtained four bands (named 1, 2, 3, and 4) from GSDNs by sucrose gradient ultracentrifugation. Among them, band 3 (spherical in shape, Figure 2c) was identified as prominent with an average diameter (as determined by DLS) of ∼344.8 nm [8]. For the band 3, the zeta potential analysis revealed the value of −25.4 mV. Baldini et al. [66] isolated CDNs by differential centrifugation and found the round- or cup-shaped objects by transmission electron microscopy (TEM). The size distribution range was from 30 to 100 nm in diameter. Seven edible mushrooms, including white bottom, Swiss brown, king oyster, shiitake, white beech, brown beech, and oyster, were characterized for the presence of exosome-like nanoparticles that were 100–140 nm in range by a NanoSight NS300 instrument and presented a sphere-shaped morphology by SEM [42] (Figure 2e). Finally, apple-derived NPs (APNPs) were shown to have a size ranging from 100 to 400 nm by measurement with a qNano using a NP200 nanopore at a 47 mm stretch [67].

Most PDNVs have a simple lipid bilayer structure, which is similar to that of the eukaryotic cellular membrane and are spherical in nature [53]. PDNVs can be fabricated into various multilayer substructures after extraction and reassembly of the lipid. Wang et al. [32] reported that GFDNs had a unique multilayer flower-like structure that could be used to deliver chemotherapeutic agents, siRNAs, and proteins to diverse cell types.

### 3.3 Biological properties

Generally, PDNVs derived from edible plants are nontoxic and nonimmunogenic as shown in many supporting studies. For example, a MTT (3-(4,5-dimethylthiazol-2-yl)-2,5-diphenyltetrazolium bromide) study on Colon-26 (mouse epithelial cell line derived from colon carcinoma) and RAW 264.7 cells (mouse macrophage-like cells) revealed that treatment with GDNs (up to 100 µg/mL) for 24 h had no effect on the viability of these cells after spectrophotometric measurement at 570 nm [44]. In the same study, electric cell substrate impedance sensing (ECIS) assay was used to prove that the integrity of the barrier function of Caco2-BBE monolayers was unaffected irrespective of the treatment with GDNs and that experiments with propidium iodide (PI)/Annexin V staining represented that the treatment with the same concentrations for 24 h had no effects on the percentage of apoptotic Colon-26 or RAW 264.7 cells [44]. These findings showed that GDNs seem to be nontoxic *in vitro*. *In vivo* toxicity evaluation considering healthy mice showed that with the administration of GDNs (0.3 mg protein/mouse) there was no significant change in colonic myeloperoxidase (MPO) activity or induction of proinflammatory cytokines (TNF-α, IL-6, or IL-1β, as assessed at the mRNA and protein levels) dosed by gavage for 7 days [44]. The colonic tissues of these mice observed no changes in hematoxylin and eosin (H&E) staining, intestinal epithelial cell (IEC) proliferation, or IEC apoptosis. Histological examination of H&E-stained heart, liver, spleen, kidney, and lung found unaffected tissues in terms of morphological or pathological changes in the GDNs gavage groups compared with controls [44]. Another MTT assay reported low cytotoxicity (more than 80% cell proliferation) on somatic cell (HEK293) and cancer cell (KB) by GDNs band 2 (20 µg/mL) after 24 h incubation [68]. In an *in vitro* study, it was revealed that ATPlite assays and PI/Annexin V staining with GFDNs treatment (up to 200 nmol lipid/mL) had no significant effect on cell proliferation or death rates of A549 (human type II pneumocytes) and CT26 cells (mouse fibroblasts derived from a colon carcinoma), compared to cationic 1,2-dioleoyl-3-trimethylammoniumpropane/dioleoylphosphatidylethanolamine (DOTAP/DOPE) liposome-treated cells after 24 h [32]. In the same report, the histological analysis of tissues from GFDN-treated mice (up to 200 nmol lipid/mL) showed no pathological change in the lung, kidney, liver, or spleen when compared with untreated mice when injected intravenously (i.v.) [32]. Another *in vitro* study (WST-1 cell proliferation assay) considering wheat-derived nanovesicles (WDNs) showed that there was no lethal effect of WDN treatment with concentrations up to 200 µg protein/mL on HDF (primary human dermal fibroblast cell line), human umbilical vein endothelial cells (HUVECs), and HaCaT cells (human keratinocyte cell line) within 3 days of the incubation and spectrophotometric measurement at 540 nm. Alongside, the cell proliferation was significantly increased in a dose-dependent manner (concentrations of 30–200 µg protein/mL up to 3 days in all cell types) [69]. Besides, the number of apoptotic cells was substantially reduced when cells were treated with WDNs, whereas there was no change in the cell cycle phase distribution, indicating that WDNs exert their proliferative effects due in part by their anti-apoptotic properties. An *in vitro* study revealed that GSDN treatment (from the interface of 45% sucrose gradient (band 3) at concentrations up to 30 µg/mL) exhibited no cytotoxicity on cells B16F10 cells (mouse melanoma), 4T1 cells (mouse mammary carcinoma), and nonmalignant HEK293 cells (human embryonic kidney) for 72 h. *In vivo* studies with mice revealed that intraperitoneal (i.p.)-injected GSDNs did not lead to any changes in blood cells, hemoglobin, and platelets. No statistically significant differences were also detected by evaluating liver enzymes, kidney function, and hematologic toxicity. H&E staining experiment revealed that no apparent organ or tissue damages in the brain, heart, kidney, liver, lungs, or spleen were observed in GSDN-administrated mice, compared with those in the control group [54]. Collectively, these studies suggest that PDNVs are very safe *in vitro* and *in vivo*.

For the use of PDNVs as a vehicle for intracellular drug delivery, it is very important to ensure the efficient uptake of nanoparticles by target cells [70]. Wang et al. [32] evaluated the GFDN uptake efficiency by different cell types. The cells were treated with PKH26-labeled GFDNs and examined by confocal microscopy or fluorescence-activated cell sorting (FACS) to analyze quantitatively. The results indicated that the majority of GL26 (mouse glioma cell line), A549, SW620 (human colon carcinoma cell lines), CT26, and 4T1 cells internalized the PKH26-GFDNs. Another study [66] revealed that PKH26-labeled CDNs were uptaken by mesenchymal stromal cells (MSCs), as examined by the fluorescence microscopy. The intracellular signals from uptaken PKH26-labeled CDNs were found after 24 h, whereas no fluorescent signal was detected in the negative control. An indirect viability assay was also carried to demonstrate the nontoxic effect of PKH26-labeled CDNs on MSCs. Another study [65] was carried out to demonstrate the uptake of B-AuNPs by breast (triple negative) cancer cell lines (MDA-MB-231) and prostate cancer cell lines (PC-3). The experiments were evaluated considering two concentrations (25 and 50 µg/mL) by dark field optical microscopy and also through the TEM image analysis at two different time points. The results showed the confirmation of internalized into both prostate and breast cancer cells and that the identity of individual nanoparticles remained intact inside the cells. APNPs uptake was observed in the human epithelial colorectal adenocarcinoma (Caco-2 cells) within 6 h by confocal microscopy [67].

Another important issue in the field of PDNVs is to see if they may alter the transport properties of intestinal epithelial cells. APNPs were considered in this context [67] and were found to decrease the expression of many colonic epithelial transports, among which organic-anion-transporting polypeptide (OATP) 2B1 is pharmacologically important for humans due to the transport of fexofenadine, an antihistaminic drug [71]. APNPs decreased OATP2B1 as both mRNA and protein and also the uptake of [^3^H] Estrone-3-sulfate. Further studies determined that APNP-derived miRNAs were internalized by Caco-2 cells and that inhibited OATP2B1 expression by binding to the 3′-UTR of its gene [67]. These results, beyond considering that PDNVs can alter the transport function of GI tract epithelial cells, offer the possibility that food-derived NPs could be used to deliver large molecules to treat diseases of the GI tract.

The main focus of the targeted therapy is to customize nanoparticles in such a way that they accumulate at the target site instead of at the disease-unrelated peripheral tissues. Hence, tissue distribution studies are an important step in designing nanoparticles and determining their potential targets [47]. Studies showed that DiR-labeled GFDNs administered by intramuscular injection were mostly localized in the muscle, and on the other hand, those applied via the intranasal (i.n.) route were located in the lung and the brain after 72 h, which was evaluated by Kodak Image Station 4000MM Pro system or the Odyssey imaging system [32]. On the other hand, the analysis by fluorescence-activated cell sorting (FACS) indicated that i.v.-injected DiR-labeled GFDNs were taken up by splenic liver cells and remained in the liver, spleen, and lung for 20 days. The particles were cleared from the kidney and the brain by days 1 and 5, accordingly [32]. Another *in vivo* study [31] was carried out to determine the tissue distribution of GDNs (both band 1 and band 2) by oral administration of (50 mg proteins) DiR-labeled nanovesicles. DiR fluorescent signals were predominantly detected in the liver and in mesenteric lymph nodes (MLNs) after 12 h of oral administration and evaluated by Kodak Image Station 4,000 MM Pro system. The fluorescent signals were not detected in the lung, spleen, or other organs. Confocal immune-staining for albumin was also used to further confirm the presence of DiR-labeled GDNs in the liver, which suggested that hepatocytes are the primary cells targeted by the nanoparticles. In another *in vivo* study [54], i.p. and i.v. administrated DiR-labeled GSDNs were detected in the liver and spleen of an experimental mouse model after 72 h. No signal was detected in the lung, heart, kidney, and brain of an experimental mouse model.

Stability of particles, including chemical stability as well as colloidal stability can change depending on the incubation time. The chemical stability is susceptible to degradation and dissolution of the particles, whereas colloidal stability is influenced by pH, ions, and macromolecules in the biological fluid [44]. There are many recent studies that have challenged the common belief that PDNVs are not stable. Wang et al. [32] reported that GFDNs are more stable than cationic liposomes DOTAP/DOPE at 37°C in the presence of 10% bovine serum. In the same study, it was mentioned that the GFDNs were very stable at 4°C for more than 1 month, and a loaded cargo (curcumin) maintained its biological activity during this period [32]. Zhang et al. [44] discovered that GDNs were very stable in stomach- and intestine-like solutions and tolerant of freeze/thaw cycles. The stability of B-AuNPs was evaluated by monitoring the plasmon (*λ*_max_) signal in various biological fluids (0.5% cysteine, 0.2 M histidine, 0.5% human serum albumin, 0.5% bovine serum albumin, and 1% NaCl solution) at different time points (1, 4, 24, 48 h, and 1 week). Stability at phosphate buffer solutions at pH 7 and 9 were also observed. The results had confirmed that the B-AuNPs demonstrated excellent stability in biological fluids at the physiological pH [65].

### 3.4 Anti-oxidant, anti-inflammatory, and regenerative activities

Exosome-like NVs from edible plants and fruit have been demonstrated to modulate the inflammatory and immune responses [33,41,42,49,53,64]. Table 2 summarizes the results of these studies. In particular, ginger, grapefruit, and broccoli NVs are able to increase anti-oxidant and anti-inflammatory mediators in macrophages, while limiting the production of proinflammatory cytokines such as TNF-α and IL-1β [41,53,64]. However, the molecular mechanisms underlying these effects are not known. Recently, nine fruit and vegetables were tested for the activity of their exosome-like NVs in respect with the nucleotide-binding domain and leucine-rich repeat containing family, pyrin domain containing 3 (NLRP3) inflammasome [33]. Grapefruit, rhizomes of ginger and turmeric, garlic cloves, leaves of cilantro, aloe vera, dandelion, lavender, and cactus stem were processed, and the derived exosome-like NVs were assessed for their effects on NLRP3 activation in bone marrow-derived macrophages (BMDMs). While the most of the NVs tested had mild inhibitory or stimulatory effects on NLRP3 activation (Caspase 1 cleavage) and downstream effects (IL-1β release), GDNs (up to 3 × 10^10^ mL) strongly suppressed both Caspase 1 cleavage and IL-1β release with a 16 h-incubation followed by a NLRP3 activation step. GDNs were easily taken up by BMDM (after a 16 h incubation time), which were inhibited in the secretion of IL-18, another cytokine dependent on inflammasome activation, and in the pyroptotic response as assessed by the release of lactate dehydrogenase (LDH). Lipids, rather than proteins and RNAs, were the active biomolecules responsible for the inhibition of NLRP3 inflammasome. Since GDNs blocked the assembly and activation of NLRP3 inflammasome, these NVs represent a new promising class of NLRP3 inflammasome inhibitors. Exosome-like nanoparticles from six mushrooms presented various effects on the NLRP3 inflammasome in BMDM [42]. Interestingly, NVs from Shiitake mushrooms (SMNs), at a concentration range of 1–9 × 10^10^ mL, remarkably inhibited both IL-1β secretion and Casp1 activation with a 16 h incubation time followed by the NLRP3 inflammasome activation step. Moreover, SMNs inhibited IL-18 secretion and suppressed LDH release. Interestingly, these NVs were able to inhibit the inflammasome activation when preincubated with BMDM before the stimulus, as lipopolysaccharide (LPS) plus sodium palmitate, or by three other activators, such as alum, nigericin, or ATP.

**Table 2:** Anti-oxidant, anti-inflammatory, and regenerative properties of plant-derived nanovesicles

Vegetable/fruit	Properties	Ref.
**Anti-oxidant and anti-inflammatory properties**		
Grapefruit	GFDNs enhance nuclear translocation of Nrf2 in RAW 264.7 macrophages	[41]
Carrot	NVs enhance expression of *IL-10*; enhance nuclear translocation of Nrf2 in RAW 264.7 macrophages	[41]
Ginger	GDNs (band 2) enhance expression of *HO-1* and *IL-10*; lower expression of *IL-6*; enhance nuclear translocation of Nrf2 in RAW 264.7 macrophages	[41]
	GDNs block NLRP3 activation in BMDM as judged by inhibition of Caspase 1 cleavage and IL-1β release	[33]
	GDNs (band 1 and band 2) increase Nrf2 nuclear translocation and reduction of ROS in mouse hepatocytes	[31]
Grapefruit	GFDNs upregulate the expression of *HO-1* and inhibit of the production of TNF-α and IL-1β in intestinal macrophages	[53]
Broccoli	BDN-derived lipids impair the ability of BMDCs to respond to LPS; they induce an anti-inflammatory response in BMDC-T cell co-cultures	[64]
Shiitake mushroom	SMNs block NLRP3 activation in BMDM, as judged by inhibition of Caspase 1 cleavage and IL-1β release, upon different inflammasome activators	[42]
**Regenerative properties**		
Grape	GELNs promote *ex vivo* intestinal stem cell proliferation and organoid structure formation	[49]
Ginger	GDNs dampen inflammation and epithelial erosion in the DSS-induced ulcerative colitis in mouse; reduce anti-inflammatory cytokines (TNF-α, IL-1β, and IL-6) and induce anti-inflammatory and pro-healing cytokines (IL-10, IL-22); accelerate wound healing in Caco2-BBE monolayers	[44]
Wheat grass	WDNs promote proliferation and exert anti-apoptotic effects on HDF (primary human dermal fibroblasts), HUVEC (human endothelial vascular endothelial cells), and HaCaT (human keratinocytes), induce tube branching in HUVEC, and increase collagen type I expression in HaCaT as both protein and mRNA	[69]

BMDCs: bone marrow-derived dendritic cells; BMDM: bone marrow-derived macrophages; HO-1: heme oxygenase-1; IL: interleukin; Nrf2: nuclear factor (erythroid-derived 2)-like 2; NVs: nanovesicles; LPS: lipopolysaccharide; ROS: reactive oxygen species; TNF: tumor necrosis factor.

**Table 3:** PDNVs as drug carrier

Source of the nanovectors/nanocarriers	Target	Drug substances	Ref.
Grapefruit	Colon cancer	Doxorubicin (Dox), curcumin	[111]
	Tumor cells (4T1, GL26, A549, CT26, or SW620)	JSI-124 (anti-Stat3 inhibitor), paclitaxel (PTX), luciferase gene siRNA	[32]
	GL26 brain tumor cells	miR17	[114]
	Intestinal macrophages	Methotrexate (MTX)	[53]
	Liver Kupffer cells	miR-18a	[112]

Broccoli	Colon tissue	Sulforaphane	[64]

Apple	Human epithelial colorectal adenocarcinoma (Caco-2) cells	miRNAs	[67]

Citrus limon	Chronic myeloid leukemia (CML)	Proteins	[55]

Ginger	Colon cancer cell lines	Doxorubicin (Dox)	[40]
	Colon-26 cells and HT-29 cells		
	RAW 264.7 macrophages and Colon-26 cells	siRNA-CD98	[113]
	Intestinal epithelial cells (IEC)	miRNA, 6-gingerol, and 6-shogaol	[44]

The nuclear translocation of nuclear factor (erythroid-derived 2)-like 2 (Nrf2) leads to the activation of a pleiotropic cytoprotective defense processes that includes antioxidant and protects against inflammatory diseases by inhibiting oxidative stress-mediated tissue injuries [72,73,74,75]. Grapefruit and ginger-derived NVs (band 2 was analyzed for ginger) can increase Nrf2 translocation to the nucleus of macrophages (RAW 264.7 cells) after a 24 h incubation time, where this transcription factor exert its cytoprotective effects [41]. Primary hepatocytes treated with band 1 and band 2 GDNs (100 µg/mL for 4 h) have also a significant increased nuclear translocation of Nrf2 and reduced production of reactive oxygen species (ROS) [31]. These effects were mediated by shogaol, confirming the previous results on the regulation of Nrf2 by shogaol-rich ginger extracts [76].

Wound healing is a multistep process that includes hemostasis, inflammation, angiogenesis, fibroblast proliferation, collagen deposition, and tissue remodeling [77]. In particular, during skin wound healing, re-epithelization involves keratinocyte proliferation and migration [78]. It is well known that plant-derived extracts and their natural compounds have demonstrated high activity in the management of wounds for different targets, such as suppressing the production of pro-inflammatory cytokines, reducing oxidative factors and enhancing anti-oxidative enzymes, and promoting neovascularization and angiogenic pathways [79]. Moreover, a number of preclinical studies have revealed that products extracted from plants can be successfully applied in modulating proliferation and differentiation of mesenchymal stem cells [80]. Furthermore, in the field of tissue engineering, plant-derived compounds or plant extracts can be incorporated into biomaterials to achieve their controlled release or used as biomaterials for cell transplantation [80,81].

A scratch assay revealed that while controls slightly closed the wound after 48 h, WDN-treated HDF, HUVEC, and HaCaT cells were found to migrate faster during the 24 h incubation period, indicating that wheat exosomes have an important role in cell migration. WDNs also promoted tube-like structures by increasing the number of branches in HUVEC seeded into matrix resembling basement membrane compared with negative controls. Both immunocytochemistry and RT-PCR analysis showed that WDNs also induced an increase in collagen type 1 (Col1) protein and mRNA expression. Although *Triticum aestivum* Linn. (wheat grass) has been shown to possess anticancer, anti-ulcer, and anti-arthritic activities [82], due to its high chlorophyll content, essential vitamins, minerals, vital enzymes, amino acids, and dietary fibers, as well as anti-oxidant properties for the presence of bioflavonoids such as apigenin, quercetin, and luteolin, further studies are needed to understand which component of WDNs from *T. aestivum* is involved in these regenerative and reparative responses.

Would healing properties of GDNs have been studies *in vitro* and *in vivo* in the context of colon inflammation [44]. The dextran sulfate sodium (DSS) model in mice was used to induce colitis with ulceration, a well-established model for the study of human ulcerative colitis. Immunocytochemistry studies revealed that, in mice sacrificed 7 days after the start of treatment, DSS had induced robust signs of inflammation, with epithelial erosion, and intestinal edema. In experiments evaluating the effects of GDNs, mice in both DSS and GDN treatment groups were first subjected to 7 days of DSS. Then, both groups were changed to regular water for an additional 7 days wound healing period; mice in the treatment group were orally administered GDNs (0.3 mg protein/mouse) every day at the same time. Interestingly, treatment with GDNs band 2, but not with GDNs band 1, prevented these signs of intestinal inflammation. Notably, the expression of E-cadherin, which contributes to maintain epithelial integrity and tissue architecture, was increased in GDN band 2-treated mice. It was also shown that orally administered GDNs dramatically decreased the levels of pro-inflammatory cytokines (TNF-α, IL-1β, and IL-6), while increasing the levels of anti-inflammatory and prohealing cytokines (IL-10, IL-22). To assess the effects of GDNs on intestinal tissue homeostasis and the ability to trigger repair mechanisms after injury, the apoptosis and proliferation of IEC were studied *in vivo* using TUNEL (terminal deoxynucleotidyl transferase dUTP nick-end labeling) assays and by staining for the proliferation marker Ki67, respectively. They found that the GDN administration reduced IEC apoptosis while increasing IEC proliferation. By using ECIS technology, it was further demonstrated that Caco2-BBE monolayers, subjected to a 30 second pulse with a frequency of 40 kHz and amplitude of 4.5 V, healed faster when cultured in the presence of GDNs (500 µL of a 0.1 mg/mL solution) compared with PBS controls (500 µL). These results were confirmed *in vivo* when mice were subjected to DSS-induced colitis for 7 days (wounding phase) and then administered with either GDNs in water or water alone for an additional 7 days (healing phase). The assessment of body weight, histological analysis, and mRNA for various cytokines demonstrated that GDNs accelerated healing of intestinal mucosal injuries.

Ju et al. [49] were the first to demonstrate that GELNs, when given by gavage (1 mg per mouse in 200 µL PBS) and imaged after 6 h, crossed the mouse intestinal mucus barrier and were taken up by Lgr5^+^ crypt stem cells by a macropinocytotic mechanism. Importantly, GELNs were resistant to degradation by the stomach acidity and the proteolytic enzymes residing along the intestinal tract. Furthermore, gavage administration of GELNs (2 mg per mouse in 200 µL PBS) every day for 7 days was shown to induce proliferation of intestinal epithelium and in particular of Lgr5^+^ stem cells that determined the increase in the stem cell-derived organoid growth *ex vivo*. This proliferation was due in part to the activation by GELNs of the Wnt/β-catenin/Tcf4 pathway as assessed by the increase in downstream genes such as AXIN-2, Cyclin D1, c-Myc, and EGFR and by nuclear migration of β-catenin.

## 4 Therapeutic effects of PDNVs

### 4.1 Therapeutic effects on inflammatory bowel diseases

IBD is composed of chronic, relapsing inflammatory disorders of the gastrointestinal tract, including Crohn’s disease and ulcerative colitis [83]. PDNVs can influence intestinal regeneration positively, show immunomodulatory properties, and protect the gut from inflammatory diseases [41,49,53]. As mentioned earlier, GELN gavage administration induced the proliferation of intestinal stem cells [49], which are crucial in epithelial cell differentiation and required intestinal tissue homeostasis and repair [84,85]. Due to these premises, the effect of orally administrated GELNs on DSS-induced colitis injury was studied. GELN treatment (2 mg/mouse/day) reduced the mortality of mice treated by DSS, i.e., within 13 days, there was 100% mortality of the control group, whereas it took 25 days for 100% mortality of the GELN-fed mice. The treatment prevented the progression of the disease as evidenced by the little reduction in the intestine length and the villus height at day 7 of administration, comparable to those of naive mice. These results were paralleled by a remarkable increase in Lgr5^+^ Ki67^+^ double-positive stem cells as well as activation of the Wnt/β-catenin pathway, indicating that GELNs accelerated mucosal epithelium regeneration and induced a rapid restoring of the intestinal architecture under DSS-induced colitis.

Further studies showed that PDNVs from ginger (band 2), carrot, grape, and grapefruit are taken up by F4/80^+^ intestinal macrophages and by Lgr5^+^ intestinal stem cells 6 h after oral administration to mice (1 mg per mouse in 200 µL PBS) [41]. However, *in vitro* studies in RAW 264.7 macrophages revealed that only ginger-derived exosomes (at 1 µg/mL and after 24 h of incubation) significantly enhanced *heme oxygenase-1* (*HO-1*) and *IL-10* expression, two genes involved in the control of oxidative stress and inflammation. The nuclear translocation of Nfr2, a key regulator of the *HO-1* gene, was induced at higher levels by ginger exosomes, followed by carrot and grapefruit. Ginger-derived exosomes (from band 2) were also able to induce the pro-inflammatory cytokine IL-6, indicating that ginger may have a role in maintaining intestinal homeostasis in terms of production of anti-inflammatory and pro-inflammatory cytokines. All four of the plant-derived exosomes were able to increase the number of Wnt/Tcf4-positive intestinal cells when orally administrated to mice (gavage administration twice a day for 3 days with 2 mg of PDNVs per mouse in 200 µL PBS). Overall, it seems that GDNs show a higher beneficial effect for maintaining gut homeostasis compared to other plants; however, further studies have to be carried out to comprehend if eating different plants may have additive or even synergic actions.

The same group produced another investigation on GFDNs and their effects on intestinal macrophages [53]. First, their physicochemical features did not change across a wide range of pH. These GFDNs were not toxic at both local and systemic levels (mice were daily given 10 mg protein/kg GFDNs for 7 days), as assessed by immune cell population and serum cytokine levels, and pretreatment with GFDNs protected mice from DSS-induced colitis, as it was previously observed for GELNs [49]. The inflammatory profile was changed by the GFDN treatment, as demonstrated by the reduction in the expression of IL-6 and Il-1β, two pro-inflammatory cytokines, as well as of MCP-1, CXCL-9, and CXCL-10, chemokines involved in the recruitment of inflammatory monocytes and T cells. GFDNs were internalized by intestinal macrophages that were demonstrated *ex vivo* to express enhanced *HO-1* and *IL-10* and to produce less IL-1β and TNF-α when isolated from mice prefed with GFDNs. To improve methotrexate (MTX) pharmacodynamics, MTX was incorporated into GFDNs (GMTX) and administered to mice in the DSS-induced colitis model. On day 3, 5, and 6 of DSS-induced colitis, mice were treated with free MTX (5 mg/kg body weight) or GMTX (MTX equaled to 5 mg/kg) by oral administration. Colon tissue damage and inflammatory cell infiltration of mice treated with GMTX were much less than those treated with free MTX or phosphate-buffered saline (PBS), while mice treated with free MTX had aggravated symptoms of colitis in comparison with PBS-treated mice in terms of degree of colon tissue damage, colon length reduction, and decreased expression of E-cadherin, an adherent junction protein involved in intestinal barrier function and homeostasis.

Intestinal immune homeostasis can be regulated by BDNs, as shown in three different models of mouse colitis [64]. In the DSS colitis model, mice were gavaged with BDNs (250 mg protein/mouse in 200 µL PBS) before (every day for 10 days) and after (every 2 days for 12 days) administration of water containing DSS. In the colitis model induced by adoptive transfer of naive T cells into Rag-1-deficient mice, BDNs (250 mg/mouse in PBS) were given orally twice every week after adoptive transfer of naive CD4+ T cells (0.5 × 10^6^, injected i.p.) and assayed 4 weeks after T cell reconstitution. Both in the DSS-induced colitis model and in the colitis model induced by adoptive transfer of naive T cells, BDNs prevented intestinal damage and determined a reduction in the expression of TNF-α, IL-17A, and IFN-γ and an increased expression of IL-10. Moreover, they lowered the number of Cd11b^+^ dendritic cells (DCs) in MLNs and colon tissue as well as their activation, inducing rather a tolerogenic profile. Another model of experimental colitis was obtained by the injection of an agonistic anti-CD40 antibody in Rag^−/−^ mice, in which the intestinal inflammation is driven primarily by macrophages and DCs in the absence of B and T cells. Mice were given BDNs orally (250 mg/mouse in PBS) every day for 10 days before injection with anti-CD40 and evaluated at day 7 after injection with anti-CD40. BDN-treated mice developed considerably milder colitis, as judged by weight loss, histological scores, and their disease activity index, compared with the PBS-treated one, suggesting that preventing DC activation is one of the cellular mechanisms underlying BDN-mediated prevention of mouse colitis. Furthermore, BDN-derived lipids (200 µM) were shown in *in vitro* studies to drive the induction of IL-10^+^ and PD-1^+^ T cells that would favor inhibition of gut inflammation, a finding suggestive that BDNs can induce tolerogenic CD11c+ DCs. Adenosine monophosphate-activated protein kinase (AMPK) was found activated in the colonic tissue of BDN-treated mice and was essential in the induction of tolerogenic DCs as well as in the protection against colitis. Among the BDN-derived lipids, sulforaphane (SFN) was further studied and shown to activate AMPK DCs *in vitro* and to protect mice from colitis when DCs treated with liposome-like nanoparticles (LNs) enriched with SFN were adaptively transferred. Conversely, LN^SFN−/−^-treated DCs were not able to reduce inflammation, strongly indicating that SFN contributes to BDN protection.

Recent findings show that PDNVs may influence gut microbiota and have a positive effect on colitis [52]. RNAs from ginger, carrot, or grapefruit PDNVs were extracted and encapsulated in PDNV-like exosomes made with PDNV-extracted lipids. Mice gavaged with PDNV RNAs (500 mg lipid/kg of body weight for three times in 7 days) exhibited a change in the composition of microbiota. In particular, ginger-derived PDNV RNAs induced several species of *Lactobacillus*, while carrot PDNV RNAs seemed to have no effect on the *Lactobacillus* level. Experiments with PA-depleted PDNV lipid-enriched vehicles demonstrated that PA is involved in the uptake of those nanovesicles by *Lactobacillus rhamnosus* and that it plays a role in maintaining the duration and the amount of PDNV accumulation in the gut. This effect was mediated by ginger-derived miRNA ath-miR167a that regulates the pilus protein SpaC mRNA and regulates the SPAC expression. Mice fed with these RNA-containing nanovectors not only were demonstrated to be taken up by *L. rhamnosus in vivo* but to give superior protection against DSS-mouse colitis compared with nanovectors transporting scrambled RNA, when given orally (500 mg/kg) daily 7 days before and 8 days after DSS administration. Interestingly, these effects were accompanied by an increase in IL-22, a cytokine involved in antimicrobial immunity and tissue repair at mucosal surfaces.

### 4.2 Therapeutic effects on liver diseases

A study evaluated tissue distribution and therapeutic effects of GDNs on alcoholic-induced liver disease [31]. GDNs (band 1) were characterized for biodistribution after oral administration to mice. DiR fluorescent signals were predominantly detected in the liver with a peak intensity at 12 h and in MLNs, whereas signals were not detected in the lung, spleen, or other organs. Confocal microscopy revealed that hepatocytes are the major site of GDN deposition. Mice were pretreated with GDNs by gavage (50 mg/mouse/day, based on protein content) or PBS as a control for 7 days and then continuously giving GDNs or PBS to the mice after they were treated with ethanol-rich diet until day 14. At the end of the treatment, mice presented less lipid droplets in the hepatocytes as well as less liver triglyceride levels and reduced liver weight compared with mice fed with alcohol alone.

Fulminant hepatic failure (FHF) can be induced in mice by the administration of d-galactosamine and a low dose of LPS (GalN/LPS), stimulating many of the clinical manifestation of FHF in humans [86]. To evaluate the protective effect of SMNs, mice were administered with PBS or SMNs (1 × 10^10^ g) using intraperitoneal injection. The mice received a GalN/LPS mixture through intraperitoneal injection 48 h later and were sacrificed after 6 h. Pretreatment of mice with SMNs alleviated pathological liver modification, i.e., hemorrhage and cell death [42]. In the serum, the levels of two downstream cytokines of NLRP3 inflammasome, IL-1β and IL-18, were significantly decreased. Importantly, the elevated serum levels of ALT and AST were mitigated by the pretreatment with SMNs.

### 4.3 Therapeutic effects on cancer

PDNVs show also interesting properties for cancer treatment. Raimondo et al. [55] have demonstrated that *in vitro*, CDNs (using 5 and 20 µg/mL for 24, 48, and 72 h) can exert an antiproliferative action on various cancer cell lines from lung, colon, and leukemia but not on normal cells in a dose- and time-dependent manner. Interestingly, these researchers have shown that CDNs are able to target one of the most important mechanisms in the cancer development: the balance between pro- and anti-apoptotic signals. Indeed, when cancer cell lines were treated with CDNs, an increase in the expression of the pro-apoptotic molecules Bad and Bax was observed. In contrast, anti-apoptotic molecules, such as Survivin and Bcl-xl, were decreased in their expression. Furthermore, *in vitro* CDNs treatment caused an increased expression of TRAIL-receptor as well as DR5 together with the increase and release of TRAIL, thus hypothesizing an autocrine loop induced by lemon vesicles that leads to the cancer cell death. To validate these results, an *in vivo* xenograft model of chronic myeloid leukemia (CML) was used. The injection of CDNs, either locally (intratumor, 50 µg per mouse per 3 days a week for 2 weeks) or i.p., resulted in the suppression of the subcutaneous tumors growth compared with PBS only-treated mice. This result was associated with both an increased expression of pro-apoptotic molecules via TRAIL/DR5 pathway and the reduced levels of pro-angiogenic cytokines VEGF-A, IL-6, and IL-8. Supposedly, these effects are mediated by miRNA, since as for mammalian cell derived-EVs, plant EVs contain microRNAs which can regulate the levels of proteins at the post-transcriptional level in animals and plants [59]. Our research group has conducted a preliminary study to test if BDNs can exert an effect on the metabolic activity, *in vitro*, on lung cancer cell lines. Interestingly, after purification by ultracentrifugation and application of a sucrose density gradient, we observed that nanoparticles with a mean diameter of 440 nm can inhibit the metabolic activity and hence the growth of two lung tumor cell lines upon their uptake (unpublished results).

#### 4.3.1 Tumor-associated macrophages as potential targets for PDNVs

Recently, it has been demonstrated that exosomes might be a useful tool in cancer immunotherapy [87,88,89]. Since monocytes/macrophages play vitally important roles in anti-infective immunity, the maintenance of tissue homeostasis, chronic inflammation, and tumors [90,91], some essential functions of monocytes/macrophages are exposed hereafter. Macrophages clear away harmful matter, including cellular debris and tumor cells *in vivo*. Moreover, macrophages mediate nonspecific defense (innate immunity) and help initiate specific defense mechanisms (adaptive immunity). In addition to stimulating the immune system, macrophages exert an immune modulatory impact by secreting various cytokines and activating the complement system, which may lead to inflammation. Macrophages are also involved in the resolution of inflammation [92,93], normal tissue development [94], and tissue repair responses to biomaterials [24]. Monocytes are recruited from the circulation into normal healthy tissues or at sites of injury, inflammation, infection, or malignancy where they then differentiate into tissue macrophages. They then acquire a distinct phenotype and activation status in response to factors present in the local tissue microenvironment. They are described as being “classically” activated by microbial products or IFN-γ to express an M1 phenotype and express high levels of proinflammatory cytokines and major histocompatibility complex molecules and are capable of killing of pathogens and tumor cells [95]. Conversely, stimulation with TH2 cytokines, such as IL-4, IL-10, and IL-13, drives macrophages toward an “alternatively” activated or M2 phenotype. In this state, they moderate the inflammatory response, promote angiogenesis and tissue remodeling, and clear cell debris [95,96]. However, more recently, the plasticity of macrophage phenotypes has been acknowledged by the subdivision of the M2 classification into M2a, M2b, and M2c subgroups according to their inducing stimuli. M2a (induced by exposure to IL-4 and IL-13) and M2b (induced by combined exposure to immune complexes and Toll-like receptor [TLR] or IL-1R agonists) exert immunoregulatory functions and drive type II responses, whereas M2c macrophages (induced by IL-10) are more related to suppression of immune responses and tissue remodeling [97]. Solid tumors consist of both malignant cells and a number of nonmalignant stromal cell types, including endothelial cells, fibroblasts, and various cells derived from the bone marrow. Complex interactions occur between these cell types within the tumor microenvironment and impact on tumor growth, progression, metastasis, and angiogenesis [98]. There is a marked myeloid cell infiltrate in most tumors, and activation of these is now known to play a key role in tumor progression [99,100]. A cell that has achieved considerable prominence in this context is the tumor-associated macrophage (TAM). Macrophages are recruited to the tumor site by the microenvironment, which produces cytokines and chemokines. It has been proposed that the recruitment and differentiation progress are related to local anoxia, inflammation, and high levels of lactic acid. The CC chemokines, such as CCL2, CCL11, CCL16, and CCL21, which are major determinants of macrophage infiltration and angiogenesis, have been demonstrated to function in the cancer of breast, lung, esophagus, ovary and cervix, and CCL2 primarily contributes to the recruitment of macrophages [101,102]. Moreover, TAMs can produce CCL2, which means that they can enlarge the recruitment of other macrophages [103]. Involved in different microenvironments, macrophages acquire different specific phenotypes [92]. The phenotypes of TAMs are plastic and regulated by the local microenvironment. Indeed, TAMs have been confirmed in recent studies to be present in large amounts in tumor tissues and to be significantly associated with the tumor development progress [104]. Strictly speaking, the division of macrophage types is complex. TAMs are not regarded as a classical subgroup of macrophages because these cells cannot be observed in the steady state but rather related to specific pathologic conditions such as inflammation and tumors. There are some special receptor tyrosine kinases consisting of TAM receptor family, including Tyro3, Axl, and MerTK, and these receptors not only are of importance in interacting with tumor cells but also play roles in macrophage polarization, efferocytosis, and autoimmune disease [105]. Active TAMs have several properties similar to M2. As a consequence, sometimes M2 macrophages are defined as TAMs in a narrow sense [106,107]. However, previous studies have shown that TAMs not only have the characteristics of M2 but also share M1 and M2 signature polarization. Therefore, the view that TAMs are equal to M2 is inaccurate [106]. TAMs have profound effects on increases in angiogenesis, tumor invasion, and the depression of immunity, and as a result, TAMs can be taken into consideration in tumor immunotherapy [108,109]. Therefore, an alternative therapeutic approach is altering/reprogramming TAMs so as to abolish phagocytes’ tumor-supportive functions and promoting their antitumor immune actions. Cao et al. [54] have demonstrated that GSDNs (10 µg protein/mL for 48 h) significantly promoted the polarization of BMDM from M2 to M1 *in vitro*. GSDNs exerted similar results *in vivo* in a mouse model of melanoma: B16F10 cells were allografted subcutaneously and after 7 days mice were treated i.p. with 250 µg/mouse every 4 days. GSDNs significantly suppressed the melanoma growth with the increased presence of M1 macrophages detected in the tumor tissue. Interestingly, this polarization depends on interaction between TLR-4 and some GSDN components such as ceramide, lipids, and proteins.

#### 4.3.2 PDNVs for delivery of antitumoral agents

In the context of cancer treatment, another important role of PDNVs might be the delivery of anticancer therapeutics, without inducing cell damage conversely to conventional liposomes. In the context of the treatment of brain tumors, it was hypothesized that GFDNs may deliver the anti-signal transducer and activator of transcription 3 (Stat3) inhibitor JSI-124 to the brain via the i.n. route and subsequently inhibit the implanted GL26 tumor growth. The rationale is to reach microglia cells, the brain resident macrophages, and dampen their pro-inflammatory and protumoral effects [110]. Intranasal administration of GFDNs encapsulating JSI-124 (12.5 pmol), but not free JSI-124 or plain GFDNs, for 10 consecutive days effectively reduced the tumor size on 15 and 20 days postinjection and prolonged the survival of mice [32].

Zhang et al. [40], in a study concerning the treatment of colon cancer, have demonstrated that doxorubicin-loaded GDNs enhanced the delivery of this drug, when GDNs were conjugated with the targeting ligand folic acid (FA). Moreover, the use of FA-GDNs was associated with a decreased systemic toxicity of the drug while extending the circulation time up to 48 h and thereby increasing its efficiency against tumors. The same authors, using a mouse model of “colitis-associated cancer” (CAC), have shown that GDNs (0.3 mg protein/mouse by gavage, every day throughout the experiment that lasted 49 days) could inhibit the tumor growth by reducing the proliferation of IEC [44]. Interestingly, this inhibition was mediated by reduction of pro-inflammatory cytokines/chemokines, including the critical regulators IL-6, IL-1β, and TNF-α. Moreover, GDN treatment was able to increase the expression of proteins usually downregulated in human colon tumors such as transgelin and cGMP-dependent kinase. Based on these promising results, two clinical trials are currently underway in the United States, one concerning the application of GELNs to prevent oral mucositis associated with chemoradiation of head and neck cancer (ClinicalTrials.gov Identifier: NCT01668849), and another focused on the use of aloe vera- and ginger-derived EVs to reduce insulin resistance and chronic inflammation in patients diagnosed with polycystic ovary syndrome (PCOS) (ClinicalTrials.gov Identifier: NCT03493984).

### 4.4 PDNVs as drug carrier

Currently, the molecular drug therapy field is limited by the lack of vehicles that permit high efficiency transfection of targeted cells without a resulting cytotoxicity or host immune response [32]. Nanotechnology can be considered in the area of drug delivery due to the ability of nanoparticles to deliver hydrophobic drugs substances as well as their superiority of targeting sites of disease. Exosomes produced by mammalian cells can be either used as such or engineered to encapsulate therapeutic agents, especially directed against cancer, presenting beneficial features, including safety, and low immunity, regulation of cell signaling, desirable negative zeta potential values, and the capacity of transferring large amount of biomolecules [18]. Like mammalian exosomes, PDNVs can be considered as superior in terms of a potential drug carrier rather than other considerable vectors (such as viral and nonviral delivery systems) where potential toxicity, tissue-specific targeting, hazardous effects, and large-scale production are some limiting factors. Considering this, nanoparticles released form plants can be a better option for targeted drug delivery as they are nontoxic, have the potentiality of being manipulated/modified for redirected targeting, capable of delivering a wide range of agents, and are able to be produced in a large scale [32]. However, the high variability of lipid composition and cargoes (proteins and miRNA) between PDNVs from different plants, and the complexity of the physiologic functions regulated by all these interacting molecules, and considering PDNVs as a drug-carrier system, the combination of loaded drugs and the cargo from the PDNVs may produce inhibitory or counteractive effects. Although further studies are needed to confirm if these events occur, one strategy to avoid these complex interactions could be to use lipids derived from PDNVs and formulate empty nanovesicles (Table 3). The particles obtained from grapefruit nanovesicle-derived lipids were very stable at 4°C for more than 1 month and did not lose their ability to carry curcumin, as well as maintained the biological function of curcumin, FA, and Zymosan A (an immune stimulator) [32]. In alternative, since the majority of the plant sources have inherent and unique compounds with physiologically relevant bioactivities, it has been proposed that target delivery would be best achieved if PDNVs would be offloaded of their inherent bioactive compounds to achieve a high loading efficiency with the bioactive compounds of interest [39].

The application of safe or harmless naturally occurring nanovectors prepared from plant-derived lipids were reported as a feasible method for drug transportation *in vivo*. As we have discussed earlier, PDNVs prepared by standard methods (sucrose gradient ultracentrifugation) are widely distributed between ∼50 and ∼1,000 nm in size between and even within species [31,41,44,61,64]. Extraction of PDNV-derived lipids and their reassembly has been indicated as a method for obtaining more uniform-sized vesicles [40,111]. The plant lipid-based drug carrier system lacked cytotoxicity or adverse effects on intestinal barrier functions, which indicated that they might be valuable for efficient drug transporting system [32]. In one study, grapefruit-derived lipid nanovectors IGFDNs (coated with inflammatory-related receptor-enriched membranes of activated leukocytes) were used to deliver 200 µg doxorubicin (Dox) at the inflamed tumor site [111]. The amount of drug released was measured spectrophotometrically at 497 nm. Such coating increased the stability and detectability of IGFDNs. According to the study, the coated IGFDNs were capable of loading different drugs including chemotherapeutic drugs, such as Dox, as well as anti-inflammatory agents like curcumin. Experiment with various mouse models of inflammation-driven disorder reported that such receptor-enriched membranes had better targeting success in comparison to plain GFDNs. The leukocyte-coated IGFDNs had increased efficiency in delivering Dox to inflammatory sites. In that case, the IGFDN–Dox combination was i.v. injected into 4T1 tumor-bearing mice, and after 24 h, tumor tissues were extracted and detected by the spectrophotometric measurement at 497 nm [111]. Teng et al. [112] reported that lipid nanovesicles derived from GFDNs and encapuslated with miR-18a were taken up by liver Kupffer cells and were visualized by confocal microscopy when assessed after 1 and 24 h of i.v. injection. GFDN-miR-18a treatment (20 nM lipids/10 µg RNA) simulated the action of M1 macrophages (F4/80^+^/IFNγ^+^ and F4/80^+^ IL-12^+^), which were responsible for the antitumor activity. At the same time, the treatment downregulated the action of M2 macrophages (F4/80^+^/TGFβ^+^ and F4/80^+^/IL-10^+^), which were dedicated to the promotion of tumor progression. After 14 days of an intrasplenic injection of CT26 colon tumor cells, the number and the size of liver metastases were reduced and the survival of the mice extended [112]. In another study, Zhang et al. [113] reported the delivery system obtained from GDN lipids, which were prepared with siRNA in a relatively uniform-sized preparation of lipid vehicles with an average diameter of 189.5 nm. GDN-derived lipid nanovectors were nontoxic (up to 200 µM lipids) when compared to commercial liposome preparation by the MTT cell proliferation assay and were successfully uptaken (15 pmol siRNA-FITC) by RAW 264.7 macrophages and Colon-26 cells, which were assessed by confocal microscopy after an 8 h incubation. Loading of siRNA-CD98 into GDN-derived lipid nanovectors caused the potential delivery of low dose of siRNA-CD98 (30 pmol) and reduced the gene expression of colonic CD98, which was evaluated by RT-PCR after 24 h and 48 h incubation. In another study, lipids from GFDNs were isolated and reassembled into nano-sized particles and coated with FA to enhance the aiming efficiency to target cells that express folate receptors [32]. It was reported that GFDNs were nontoxic both *in vitro* (up to 200 nmol lipids/mL considered in ATPLite assay) and *in vivo* studies (100 nmol lipids by i.v. injection into mice for 5 days) and could be used with hydrophobic agents including curcumin, FA, and Zymosan A without altering the biological activities of the agents. GFDNs with FA were used for transporting the chemotherapeutic drug paclitaxel (PTX) (200 nmol lipids/5 µg FA/20 mg/kg of body weight of PTX) i.v. to the tumor location, which thereby reducing the tumor volume in two tumor xenograft models, including the mouse CT26 cells and SW620 cells, which was visualized by using a Kodak Image System after 30 days of treatment [32]. The study by Zhang et al. [40] has been already mentioned for the activity of g GDNs against colon cancer. They also demonstrated that DiL-labeled GDNs (100 µmol lipids/L) were successfully internalized by Colon-26 and HT-29 cells via the phagocytosis pathway after a 4 h incubation as detected by the flow cytometry analysis and were nontoxic in *in vitro* (by the MTT assay using up to 200 µmol lipids/L and detected after 48 h incubation) and *in vivo* studies (by i.v. injection of 200 µmol lipids/L and measurement by H&E staining after 7 days). Encapsulation of chemotherapeutic drug Dox into GDNs (13 µmol/L Dox) was reported to prolong the release of Dox from GDNs and started rapid apoptosis, which was quantified by PI/Annexin V apoptosis assay. Collectively, the combination of reassembled lipids with therapeutic drugs could be a promising drug delivery system with less toxicity and adverse side effects of free drug substances.

PDNVs can be loaded with a variety of cargo molecules, such as small molecular drugs, siRNAs, DNA expression vectors, and proteins, directing them to different tissues [32,40,113]. siRNAs and DNAs, highly negative charged molecules, are less encapsulated into PDNVs than positively charged therapeutics [80]. However, one study reported the potential of coated GFDNs to deliver miRNA. GFDNs coated with FA and added with polyethylenimine (PEI)/RNA (FA-pGFDNs) were used to deliver RNA efficiently to the brain through an intranasal route. FA was used to enhance the targeting to GL26 brain tumor cells that express folate receptors (FRs), and PEI was considered for their high efficiency of carrying RNA and DNA. The combination was also less toxic than conventional PEI vector. Mice were treated with FA-pGFDN/miR17 (20 µg miRNA) every 3 days for 21 days beginning on day 5 after GL26 cells were intracranially implanted. The effects of the treatment were evaluated by immune staining of postfixed brain tissue with anti-DX5, luciferase, and MHCI antibodies [114]. The induction of DX5^+^ NK cells was correlated with a decrease in the expression of MHCI^+^ luciferase^+^ GL26 cells, indicating that coated GFDN/miR17 is selectively taken up by GL26 cells and subsequently inhibits the expression of MHCI expressed on the GL26 tumor cells (a target of miR17), which triggers the activation of NK cells to kill tumor cells. Mice intranasally treated with FA-pGFNV/miR17 showed also slowed growth of GL26 cells [114].

In an interesting study, *in vivo* tolerogenic effect of SFN contained in BDNs (BDN-SFN) was studied in DSS-induced mouse acute colitis [64]. Both knockout and knockin strategies were used to specify the effects of BDN-SFN. The results showed that BDN-SFN combination enhanced the effect of BDNs by histological scores and less reduction in the colon length in comparison with SFN knockout strategy. It was also reported the contribution of SFN on the *in vivo* migratory ability of DCs by the lower numbers of CD11b^+^ DCs in the MLNs and colon of treated mice [64].

As mentioned earlier, the selective uptake of GFDNs by intestinal macrophages was reported [53]. In the study, GFDNs improved DSS-induced mouse colitis. Upregulation of heme oxygenase-1 (HO-1) expression and constraining TNF-α and IL-1β production in intestinal macrophages could be explained as the influence of GFDNs. Encapsulation of an anti-inflammatory drug, methotrexate (MTX; 5 mg/kg body weight), into GFDNs (25 mg proteins in 3 mL PBS pH 7.4) showed less toxic effect and higher curative effects than free MTX for DSS-induced colitis in mice by H&E staining. According to the study, GFDNs could be a better option as intestinal immune modulator that elevate homeostasis of intestinal macrophages. The study enhanced the opportunity of using such nanoparticles as oral transportation systems for small drug biomolecule for reducing the inflammatory reactions in humans [53].

## 5 Concluding remarks

Once discovered that plant-derived nanovesicles can mediate interspecies communication, these natural derivatives are at the forefront of medicine and drug delivery. Since PDNVs interact closely with the GI tract, and thus, they are absorbed to be delivered first to the liver and other organs, the knowledge about their interaction with intestinal epithelial cells, including stem cell populations, and immune cells has been thoroughly investigated. Overall, PDNVs are readily internalized by mammalian cells and can produce distinguished effects on the functional behavior of these cell types. Although phytochemicals have been shown to effectively foster tissue regeneration and repair, the application of PDNVs to these fields is still empirical and should be further investigated at the level of different stem cell types and appropriate models. Based on their anti-oxidant, anti-inflammatory, and regenerative properties, it is expected that PDNVs may be applied in the future as therapeutic agents for chronic inflammatory disorders, such as those affecting the lung and the central nervous system. Recent evidences demonstrate that PDNVs can deliver nucleic acid as well as drugs with no overt cytotoxicity make them in a privileged position in the pharmaceutical arena applied to medical sciences. Besides miRNAs, PDNVs have been shown to deliver secondary metabolites, such as 6-gingerol and 6-shogaol from ginger, and sulforaphane from broccoli, expanding their possible role in therapeutic applications. Because PDNVs are more biocompatible than synthetic vectors and can be produced economically on a large scale, nanovehicles from edible plants represent one of the safest and most cost-effective therapeutic delivery platforms. The immunomodulatory property of PDNVs in modifying the tumor microenvironment, including macrophages, is a new frontier, which should be further explored. The limits of PDNVs are represented by their unknown mechanism of action and the many biomolecules presented by them, which may incite side effects due to the pleiotropic nature of these ingredients. Another open issue is if there any evidence that the part of the source plant makes a difference. We do not know whether nanovesicles from root, stalk/stem, or leaf differ from each other or they can considered equivalent, and thus, this issue merits attention in future studies. Finally, there is the need of further and detailed studies about PDNV pharmacokinetics, pharmacodynamics, and safety in broad animal models of disease before they can be introduced in the clinical setting.

## Abbreviations

A549 cells: human type II pneumocytes cells
AMPK: adenosine monophosphate-activated protein kinase
APNPs: apple-derived nanoparticles
AFM: atomic force microscopy
B16F10 cells: mouse melanoma cells
B-AuNPs: broccoli phytochemicals-coated gold nanoparticles
BDNs: broccoli-derived nanovesicles
BMDM: bone marrow-derived macrophages
Caco-2 cells: human epithelial colorectal adenocarcinoma
CCDNs: exosome-like nanoparticles from coconut water
CDNs: *Citrus lemon*-derived nanovesicles
CFDNs: *Citrus clementina* fruit juice-derived nanovesicles
Colon-26: mouse epithelial cell line derived from colon carcinoma
CT26: mouse fibroblast cell line derived from colon carcinoma
DCs: dendritic cells
DLS: dynamic light scattering
DOPE: dioleoylphosphatidylethanolamine
DOTAP: 1,2-dioleoyl-3-trimethylammoniumpropane
Dox: doxorubicin
DSS: dextran sulfate sodium
ECIS: cell-substrate impedance sensing
EPR: enhanced permeation and retention
ECM: extracellular matrix
EVs: extracellular vesicles
FA: folic acid
FACS: fluorescence-activated cell sorting
FHF: fulminant hepatic failure
GDNs: ginger-derived nanovesicles
GI: gastrointestinal
GSDNs: ginseng-derived nanovesicles
GELNs: grape exosome-like nanoparticles
GFDNs: grapefruit-derived nanovesicles
GL26: mouse glioma cell line
HaCaT: human keratinocyte cell line
HDF: primary human dermal fibroblast cell line
H&E: hematoxylin and eosin
HO-1: heme oxygenase-1
HUVEC: human umbilical vein endothelial cells
IBD: inflammatory bowel disease
IEC: intestinal epithelial cell
IL: interleukin
i.n.: intranasal
i.p.: intraperitoneal
ISEV: international society for extracellular vesicles
i.v.: intravenous
LDH: lactate dehydrogenase
LNs: liposome-like nanoparticles
LPS: lipopolysaccharide
MAPK: mitogen-activated protein kinase
MBVs: matrix-bound nanovesicles
MDA-MB-231: breast (triple negative) cancer cell lines
MLNs: mesenteric lymph nodes
MPO: colonic myeloperoxidase
MSCs: mesenchymal stromal cells
mTOR: mammalian target of rapamycin
miRNA: microRNA
MS: mass spectrometry
MTX: methotrexate
MTT: 3-(4,5-dimethylthiazol-2-yl)-2,5-diphenyltetrazolium bromide
MVs: microvesicles
NLRP3: nucleotide-binding domain and leucine-rich repeat containing family, pyrin domain containing 3
Nrf2: nuclear factor (erythroid-derived 2)-like 2
OATP: organic-anion-transporting polypeptide
PA: phosphatidic acids
PBS: phosphate buffered saline
PCOS: polycystic ovary syndrome
PDNVs: plant-derived nanovesicles
PI: propidium iodide
PC-3: prostate cancer cell lines
RAW 264.7 cells: mouse macrophage-like cells
ROS: reactive oxygen species
SEM: scanning electron microscopy
SFN: sulforaphane
siRNA: small-interfering RNA
SLNs: solid lipid nanoparticles
SMNs: shiitake mushroom-derived nanovesicles
SW620: human colon carcinoma cell lines
TAMs: tumor-associated macrophages
TEM: transmission electron microscopy
TNF: tumor necrosis factor
TUNEL: terminal deoxynucleotidyl transferase dUTP nick-end labeling
WDNs: wheat-derived nanovesicles
4T1 cells: mouse mammary carcinoma cells
